# Required Longitudinal Service-Learning and Its Effects on Medical Students’ Attitudes Toward the Underserved

**DOI:** 10.1007/s40670-021-01350-7

**Published:** 2021-06-18

**Authors:** Monica Rose Arebalos, Faun Lee Botor, Edward Simanton, Jennifer Young

**Affiliations:** University of Nevada Las Vegas School of Medicine, Las Vegas, NV 89102 USA

**Keywords:** Medical education, Community-oriented, Ethics/attitudes, Public health

## Abstract

Although medical students enter medicine with altruistic motives and seek to serve indigent populations, studies show that medical students’ attitudes towards the undeserved tend to worsen significantly as they go through their medical education. This finding emphasizes the need for medical educators to implement activities such as service-learning that may help mitigate this negative trend.

All students at the University of Nevada Las Vegas (UNLV) School of Medicine are required to participate in longitudinal service-learning throughout medical school, and a majority of students interact with the underserved at their service-learning sites. Using the previously validated Medical Student Attitudes Towards the Underserved (MSATU), independent sample T-tests showed that students who interact with underserved populations at their sites scored with significantly better attitudes towards the underserved at the end of their preclinical phase. Subjects included 58 medical students with 100% taking the MSATU. This result indicates that longitudinal service-learning, particularly when it includes interaction with the underserved, can be one method to combat the worsening of medical students’ attitudes as they complete their medical education.

## Introduction

The USA is currently challenged by striking disparities in health, and the growing diversity in US society has supported the need for culturally competent medical professionals [1]. Although many students enter medical school hoping to reach indigent populations, studies continue to show that medical students’ attitudes towards the underserved worsen significantly as they complete their medical education [2–7].

One intervention that may help mitigate this pattern is service-learning. Service-learning is defined as an “educational experience in which students participate in an organized service activity that meets identified community needs” and includes reflection on the service in order to gain an enhanced sense of civic responsibility [8]. A systematic review found considerable variability in program types with various names including service-learning, community-oriented learning, and community-based learning. Despite these variations, there is still evidence that service is beneficial for both the community and the medical students [9]. For many institutions, the goal of service-learning is for students to learn about population health and to promote and improve public health skills [10]. Many studies have shown that service activities are correlated with higher levels of empathy, better understanding of community needs, and an increased belief that physicians have a responsibility to provide medical care for the needy [11–15]. Additionally, when studies specifically compare groups of students who participate in some type of community service to those that do not, results suggest that attitudes towards the underserved are consistently better in service groups [16–19]. The Liaison Committee on Medical Education has recognized the potential positive implications of service-learning and has added a mandate to their standards that medical schools “provide sufficient opportunities for, encourage, and support medical student participation in service-learning and/or community service activities” [20].

While studies show promising positive outcomes of service-learning, most activities tend to be short-term or voluntary for students. None of the authors are aware of any medical school in the USA where service-learning is required across multiple years of the curriculum for all students. In an attempt to train compassionate physicians who will “understand and value all cultural aspects of medicine” [21], the University of Nevada Las Vegas (UNLV) School of Medicine became one of the few medical schools to require all students to participate in longitudinal service-learning throughout their doctorate education. As a new school, UNLV has partnered with over 50 organizations as community service sites. Shortly after beginning medical school, all students select a service-learning site. During the preclinical phase, students have scheduled time to complete at least 4 hours of service each month. During the clinical phase, service activities are more sporadic as they can fit within clerkship schedules. Students also participate in written reflections and continuous collaboration with their organization regarding goals and projects.

The purpose of this study is to analyze if required longitudinal service-learning influences students’ attitudes towards the underserved during the preclinical phase. Additionally, the purpose is to examine possible differences in attitudes between students who work directly with the underserved versus those that do not. Our hypothesis is that the results of a validated survey will demonstrate that required service-learning mitigates some of the decrease of attitudes towards the underserved, especially in students who work closely with underserved populations.

## Methods

### Instruments

The Medical Student Attitudes Towards the Underserved (MSATU) is a validated self-reported measure of medical students’ attitudes with regard to providing medical care to underserved populations. This measure consists of three subsections related to healthcare services, access to care, and US influences. The first section focuses on the responsibility of various groups (medical students, physicians, the government, etc.) in providing healthcare services to patients in indigent populations. The second section surveys the student’s opinion on whether patients should have access to various types of medical care services. The third section surveys students’ perception on how being a patient in the USA influences various aspects of medical care. The MSATU elicits responses on a 5-point Likert scale (1 = strongly disagree, 5 = strongly agree) [3, 6, 16]. We used all three subsections to calculate each students’ total MSATU score.

### Data Collection

All medical students in the class of 2021 (n = 58) received the MSATU prior to medical school and at the end of the 18-month preclinical phase. The survey was a confidential online survey students took at a time and location of their choosing.

Students were classified into two groups: those who interact with the underserved at their service-learning sites (n = 46) and those who do not (n = 12). Classification of groups was performed by the course director at the end of the preclinical phase based on organizations’ missions and self-reported activities. The sites that provide direct interaction with the underserved do so through activities such as mentorship, advocacy, or tutoring. Some examples of sites that include interaction are the following: Boys and Girls Clubs or other after-school programs, Rape Crisis and Human Trafficking Intervention, and Big Brothers Big Sisters. Sites without direct interaction with the underserved focused on a social determinant issue such as transportation, food insecurity, or housing. Students in this group augmented the organization’s internal activities and programs or conducted outreach with the general public rather than directly interacting with the underserved. Students selected their service sites and thus made their own decision regarding the amount of interaction they would have with the underserved.

### Analysis

Mean scores were calculated for each of the three subsections and the total MSATU. Changes in attitudes were calculated by subtracting the pretest score from the post-test score for each participant. Independent sample t-tests were used to examine differences between the experimental groups (those who interacted with the underserved, and those who did not). SPSS version 26.0 was used for all statistical analyses.

## Results

Fifty-eight students completed the MSATU, yielding a 100% completion rate. Regarding sociodemographic characteristics, students in this sample were 29 males and 29 females with 15 being considered underrepresented minority students (25.9%).

As demonstrated in Tables 1 and 2, students who interacted with the underserved in their service-learning began with very similar pretest scores but showed significantly less decrease in attitudes toward the underserved on the healthcare services subsection of the MSATU. While students who selected service sites that did not involve interaction with the underserved appeared to score lower on most of the pretest subscales, these differences were not statistically significant.

**Table 1:** Means and standard deviations of MSATU subscales and totals by group

		Interacted with underserved (N = 46)		No underserved interaction (N = 12)	
		Mean	SD	Mean	SD
Pretest	Healthcare services subscale	4.037	0.389	3.941	0.279
	Access to care subscale	4.380	0.691	4.097	0.630
	USA influence subscale	4.279	0.560	4.010	0.566
	Total MSATU	4.231	0.458	4.015	0.465
Post-test	Healthcare services subscale	3.993	0.421	3.698	0.547
	Access to care subscale	4.292	0.712	4.214	0.589
	USA influences subscale	4.310	0.540	3.917	0.883
	Total MSATU	4.199	0.426	3.943	0.600
Change	Healthcare services subscale	−0.044	0.286	−0.243	0.316
	Access to care subscale	−0.087	0.472	0.118	0.321
	USA influence subscale	0.031	0.577	−0.093	0.802
	Total MSATU	−0.032	0.299	−0.073	0.360

**Table 2:** Levene’s test for equality of variances

		Levene’s test for equality of variances		t-test for equality of means					95% Confidence interval of the difference	
		F	Sig	t	df	Sig. (2-tailed)	Mean diff	Std. error diff	Lower	Upper
Health care service subscale	Equal variances assumed	0.593	0.445	2.099	56	0.040	0.19880	0.09473	0.00903	0.38858
	Equal variances not assumed			1.977	16.025	0.065	0.19880	0.10053	−0.01429	0.41190
Access to care subscale	Equal variances assumed	0.853	0.360	−1.416	56	0.162	−0.20467	0.14456	−0.49427	0.08492
	Equal variances not assumed			−1.767	24.946	0.089	−0.20467	0.11583	−0.44325	0.03390
USA influence subscale	Equal variances assumed	2.585	0.113	0.612	56	0.543	0.12442	0.20337	−0.28298	0.53182
	Equal variances not assumed			0.504	14.101	0.622	0.12442	0.24673	−0.40441	0.65325
Total MSATU	Equal variances assumed	0.664	0.419	0.398	56	0.692	0.04033	0.10120	−0.16241	0.24306
	Equal variances not assumed			0.357	15.197	0.726	0.04033	0.11298	−0.20021	0.28086

## Discussion

Our students’ attitudes toward the underserved were shown to remain stable when they interact with underserved at their service-learning site, rather than worsen significantly as previous studies have shown is typical in undergraduate medical education. Although we hypothesized that all students who participate in service-learning would benefit, the discovery that only students who interact with the underserved demonstrated relatively stable attitudes was an important finding. This implies that educators who begin to implement required service-learning should consider ensuring their students that they will be interacting with underserved communities.

While the worsening of medical students’ attitudes toward indigent populations is a recognized problem in medical education, not all medical schools are surveying their students in order to measure the extent of the problem or its progression over time. In fact, in many aspects of medical school, evaluations of students’ knowledge, skills, and attitudes are done in a variety of ways, many of which are not standardized [22]. Implementing validated measures such as the MSATU will be an important step in reaching the goal of training socially responsible physicians. By measuring their students’ attitudes, educators will be able to evaluate if interventions such as service-learning are necessary and can evaluate the effectiveness of the interventions.

Despite the fact that all of our students participate in longitudinal service-learning, the actual interaction with underserved populations appears to be the most important component of this innovation. Medical science educators who hope to improve their students’ attitudes toward indigent populations, and possibly help their students remember their initial philanthropic motives for entering medicine, should consider implementing service activities in their curricula that include meaningful interactions with underserved populations.

## Conclusion

Medical students’ attitudes toward the underserved tend to worsen as they complete 4 years of medical school. This is a significant problem in medical education that will require multidisciplinary innovations on multiple levels, and the implementation of service-learning with the underserved may be an important component of the solution. The purpose of our study was to evaluate if required longitudinal service-learning was beneficial in mitigating these worsening attitudes. While all medical students were required to participate in longitudinal service-learning, only those who worked directly with underserved communities showed stable and significantly more positive attitudes than their counterparts who did not interact with the underserved. These findings can help guide medical science educators in creating meaningful experiences that help their students maintain the altruistic attitudes that they start medical school with, and to become the compassionate physicians they desire to be.

## Limitations of the Study

The UNLV School of Medicine at its foundation is designed to be geared toward community service, and students are aware of this as they apply. It is possible that students who elect to come to UNLV may be different from medical students nationwide in their focus on community service.

Before students start volunteering at their service-learning sites, they begin medical school with an introductory block in which they, in part, learn about various population health topics while completing participatory community learning activities in an assigned underserved area. All students also create a presentation about population health topics that they have witnessed in their assigned areas struggling with. It is possible that beginning medical school with this emphasis on population health may prime students to be more community-oriented and bring their altruistic motives into focus before they begin their longitudinal service-learning activities.

This study is based on a single cohort of 58 students only in the preclinical phase at a new medical school. Future studies will look at different kinds of service-learning over all 4 years of the medical curriculum. Due to the service-learning requirements, we do not have a control group of students without any service-learning exposure to compare to. Also, future cohorts may react differently to the service-learning activities.
